# Factors associated with mechanical ventilation longer than 24 h after liver transplantation in patients at risk for bleeding

**DOI:** 10.1186/s12871-023-02321-8

**Published:** 2023-11-02

**Authors:** Marta Caballero, Antoni Sabate, Lourdes Perez, Julia Vidal, Enric Reverter, Rosa Gutierrez, Gonzalo Crespo, Judith Penafiel, Annabel Blasi

**Affiliations:** 1 Department of Anaesthesiology, University Hospital of Bellvitge, University of Barcelona Health Campus, IDIBELL, Barcelona, Spain; 2 Department of Anaesthesiology, Clinic Hospital, University of Barcelona Health Campus, IDIBAPS, Barcelona, Spain; 3 Department of Hepatology, Hospital Clínic, Barcelona, IDIBAPS Spain; 4 Department of Anaesthesiology, University Hospital of Cruces, Bilbao, Spain; 5 Department of Hepatology, Liver Transplant Unit, Hospital Clínic, Barcelona; University of Barcelona; IDIBAPS; CIBERehd, Barcelona, Spain; 6 Biostatistics Unit (UBiDi), University of Barcelona Health Campus, IDIBELL, Barcelona, Spain

**Keywords:** Blood components, Liver transplantation, Mechanical ventilation, Patient outcome, Surgical Intensive care unit

## Abstract

**Background:**

This risk analysis aimed to explore all modifiable factors associated with prolonged mechanical ventilation (lasting > 24 h) after liver transplantation, based on prospectively collected data from a clinical trial.

**Methods:**

We evaluated 306 candidates. Ninety-three patients were excluded for low risk for transfusion (preoperative haemoglobin > 130 g.l^−1^), and 31 patients were excluded for anticoagulation therapy, bleeding disorders, familial polyneuropathy, or emergency status. Risk factors were initially identified with a log-binomial regression model. Relative risk was then calculated and adjusted for age, sex, and disease severity (Model for End-Stage Liver Disease [MELD] score).

**Results:**

Early tracheal extubation was performed in 149 patients (84.7%), and 27 patients (15.3%) required prolonged mechanical ventilation. Reoperations were required for 6.04% of the early extubated patients and 44% of patients who underwent prolonged ventilation (*p* = 0.001). A MELD score > 23 was the main risk factor for prolonged ventilation. Once modifiable risk factors were adjusted for MELD score, sex, and age, three factors were significantly associated with prolonged ventilation: tranexamic acid (*p* = 0.007) and red blood cell (*p* = 0.001) infusion and the occurrence of postreperfusion syndrome (*p* = 0.004). The median (IQR) ICU stay was 3 (2–4) days in the early extubation group vs. 5 (3–10) days in the prolonged ventilation group (*p* = 0.001). The median hospital stay was also significantly shorter after early extubation, at 14 (10–24) days, vs. 25 (14–55) days in the prolonged ventilation group (*p* = 0.001). Eight patients in the early-extubation group (5.52%) were readmitted to the ICU, nearly all for reoperations, with no between-group differences in ICU readmissions (prolonged ventilation group, 3.7%). Conclusion.

We conclude that bleeding and postreperfusion syndrome are the main modifiable factors associated with prolonged mechanical ventilation and length of ICU stay, suggesting that trials should explore vasopressor support strategies and other interventions prior to graft reperfusion that might prevent potential fibrinolysis.

Trial Registration.

European Clinical Trials Database (EudraCT 2018–002510-13,) and on ClinicalTrials.gov (NCT01539057).

## Background

Early tracheal extubation in liver transplantation implies a shorter stay in a postoperative ICU[1] and the avoidance of the side effects of mechanical ventilation on splanchnic blood flow, which is detrimental to the liver graft. However, adequate liver function and the absence of bleeding or other adverse conditions that may require a return to the operating theatre cannot always be ascertained at the end of surgery [2]. Furthermore, most gross haemodynamic and respiratory disturbances, bleeding or hepatic artery thrombosis can appear in the first 24 h after a liver transplant [2]. Among retrospective series from the early 2000s, immediate reintubation was necessary in 11.7% of cases [3]. That rate would be unacceptable today. In some series fast-track extubation was performed in patients with low Model for End-Stage Liver Disease (MELD) scores (around 12) [4, 5], but as waiting lists have come to include older and more overweight patients, average MELD scores have risen in recent series [6]. In this scenario, anaesthesiologists have developed an interest in finely tuning fast-track protocols in keeping with risk for reintubation and ICU readmission and, if possible, reducing that risk.

We aimed to explore all modifiable preoperative and intraoperative risk factors associated with a need to maintain mechanical ventilation for more than 24 h after liver transplantation in a multicentre series of recipients registered prospectively for a randomised clinical trial.

## Methods

Data from a multicentre, haemoglobin-stratified, randomised controlled trial on fibrinogen infusion and blood product requirements by our group [7] were used for this secondary analysis, which was foreseen in the initial protocol registered in the European Clinical Trials Database (EudraCT 2018–002510-13,) and on ClinicalTrials.gov (NCT01539057). The protocol was approved by the institutional review board (IRB) of the lead hospital (University Hospital of Bellvitge, approval number AC 033/18) as well as the IRBs of the other participating centres (University Hospital of Cruces and Clinic Hospital of Barcelona). Patients were enrolled if they gave their written informed consent.

### Patients

All adults who were scheduled for liver transplantation were assessed for eligibility from 2 August 2019 to 2 November 2021. Exclusion criteria were low risk for intraoperative transfusion (preoperative haemoglobin > 130 g.l^−1^) or high risk for intraoperative transfusion (patients on anticoagulation therapy and with bleeding disorders). Also excluded were patients whose indication for transplantation was familial polyneuropathy or who were undergoing an emergency procedure.

### Graft and anaesthesia management, surgery, and transfusion protocols

Organ recovery from controlled cardiac-death donors met the acceptance criteria established by the Spanish Liver Transplantation Society in all centres [8]. Those criteria stipulate normothermic regional perfusion in the recovery of organs from non-living donors.

The anaesthesia protocol was monitored to ensure consistency and compliance across all the research centres. Swan-Ganz catheterization was used for in-procedure monitoring, and patients with echocardiographic abnormalities at baseline additionally underwent transesophageal echocardiography. Vena cava preservation, with or without a portacaval shunt depending on the surgeon's preference, was attempted in all patients. Crystalloid fluid replacement (2 mL/kg/h) was used to maintain blood volume. Sodium bicarbonate 1/6 M was given to maintain pH 7.3. Intravenous calcium was administered to keep the plasma calcium ion concentration within the ranges of reference stipulated by each hospital’s laboratory. Normothermia was maintained. The liver allograft was preserved in University of Wisconsin solution.

Prior to reperfusion, the graft was flushed with 1000 mL Hartmann’s solution at 38 °C to remove air and detritus from the wall of the graft’s inferior vena cava. Patients were placed in the Trendelenburg position. Next, the distal end of the donor’s vena cava was closed with a vascular stapler. We used a modified definition of postreperfusion syndrome as outlined by Aggarwal et al.,[9] namely, a 30% or more decrease in blood pressure or heart rate from baseline for more than 1 min within 5 min of reperfusion of the liver graft that required additional compensatory measures such as vasoconstrictor drugs or rapid fluid infusion.

Blood product infusion criteria were as follows: red blood cells (RBCs) to maintain a haemoglobin level of > 80 g.l^−1^, platelet concentrates if a count fell to < [30,000 × 10^–9^]^−1^, and intravenous tranexamic acid boluses of 500 mg if fibrinolysis (> 15% lysis at 60 min) was detected by thromboelastometry for fibrin tissue. Cell saver devices were not used. Haemostatic management was also guided by thromboelastometry. In case of massive bleeding (> 150 ml.min^−1^), we monitored maximum clot firmness by extrinsic thromboelastometry amplitude at 10 min. If we detected a value of < 15 mm or a clotting time > 300 s, we simultaneously transfused 4 units of RBCs, 1 g of tranexamic acid, 2 g of fibrinogen concentrate, 1 unit of apheresis platelets, and 15 ml.kg^−1^ of fresh frozen plasma.

### Weaning process and tracheal extubation

At the end of surgery, a propofol infusion was started to ensure sufficient sedation for patients to tolerate the tracheal tube. On arrival to the ICU, the patients were connected to mechanical ventilation with a starting fraction of inspired oxygen (FiO2) of 50%, a driving pressure of 15 cmH2O, and a positive-end expiratory pressure of 5 cmH2O. Haemodynamic stability was checked by assessing systolic blood pressure (> 110 mm Hg) and heart rate (< 100 beats per minute), and when favourable respiratory values (oxygen saturation > 95% with FiO2 < 50%) were achieved, the propofol infusion was stopped. Once patients regained full consciousness and spontaneous ventilation was maintained with a respiratory rate < 25 breaths per minute, normocapnia without acidosis, oxygen saturation > 95% with FiO2 < 50%, and absence of bleeding, tracheal extubation was performed.

### Primary outcome, other outcomes of interest, and risk factors

The primary outcome was the need for mechanical ventilation for more than 24 h, used as a definition of extubation failure. Although postoperative respiratory failure has been defined as the need for mechanical ventilation for longer than 48 h in a large study of patients undergoing non-cardiac surgery, the study did not include liver transplant recipients [10].

Variables considered as possible risk factors included recipient and donor characteristics and data collected during surgery. Recipient characteristics were age, sex, BMI, diabetes mellitus, hypertension, cardiac disease, respiratory disease, indication for transplantation, MELD score, Child score, hospitalization when the procedure was scheduled, haemoglobin and creatinine levels, glomerular filtration rate, plasma fibrinogen levels, prothrombin time and the international normalised ratio, platelet count, and baseline thromboelastometry profile. Donor characteristics were type of donor (after brain or cardiac death) and cold ischaemia time. Intraoperative data were surgical time; warm ischaemia time; infusions of blood components, fibrinogen concentrate, tranexamic acid, crystalloid, and albumin; and the development of postreperfusion syndrome.

During liver transplantation and in the following 90 days, we recorded the incidence of intra- and postoperative thrombotic events in the graft or legs (assessed by Doppler ultrasound), and in the lung (assessed by computed tomography). Reoperations after 24 h or admission to the ICU for any cause were also recorded.

The data monitoring committee reviewed all adverse events, and an annual safety report was sent to the Spanish Agency for Medicines and Medical Products and the IRBs that approved the protocol.

### Statistical analysis

Descriptive statistics for patients and surgeries were expressed as mean (SD) for discrete variables and median (IQR) for continuous variables. Categorical variables were expressed as number of cases and percentage. Statistics related to actuarial patient mortality and graft survival were also compiled.

We used a log-binomial regression model to evaluate the associations between the risk factors and prolonged mechanical ventilation (> 24 h). Risk was adjusted for age, sex, and MELD score based on their positive association not only with the outcome (dependent) variable but also with other modifiable variables because we detected that there was substantial interaction when analysing the data. Relative risk and 95% CIs were also calculated. All analyses were performed with the statistical software package R, version 4.1.0 for Windows (http://www.R-project.org, The R Foundation).

## Results

During the period of the trial, 306 candidates were evaluated, and 93 patients were excluded because their baseline haemoglobin was > 130 g.l^−1^. Thirty-one patients were excluded for the other criteria listed above. A total of 182 patients were enrolled. After 6 procedures were cancelled, 176 patients were finally included in the analysis.

In 149 patients (84.7%), the trachea was extubated early (< 24 h). The remaining 27 patients (15.3%) required > 24 h of mechanical ventilation. Nine patients in the early extubation group (6.04%) required reoperation. Twelve patients who underwent prolonged mechanical ventilation (44.4% of the 27 patients in that group) also required reoperation (*p* = 0.001).

Patient’s characteristics for all patients and in the two assigned groups are shown in Table 1. Diagnoses of cirrhosis, partial portal vein thrombosis, plasma sodium and creatinine values, MELD scores, and haemoglobin and coagulation and thrombelastometry profiles were different between the groups. Echocardiographic abnormalities were observed in 16.50% of patients, but there were no between-group differences. The preoperative echocardiogram indicated some degree of pulmonary hypertension in 44% of patients with early extubation vs. 22% of those requiring prolonged mechanical ventilation. Angiography confirmed the presence of pulmonary hypertension in two patients in the early extubation group and one patient in longer mechanical ventilation group. Baseline PO2 ≤ 80 mm Hg, was found in 11.4% of patients in the early extubation group vs. 15% in the prolonged mechanical ventilation group. Some degree of pulmonary dysfunction was found in the preoperative computerized tomography scans in both groups, with normal function observed in 32% of those extubated early vs. 44% of those requiring mechanical ventilation for longer than 24 h.

**Table 1:** Patient characteristics Data are number (%) or percentage of patients, unless otherwise indicated as mean (SD)^a^, median (IQR)^b^, or median (range)^c^

	**All** **(*n* = 176, 100%)**	**MV > 24 h** **(*n* = 27, 15.3%)**	**MV < 24 h** **(*n* = 149, 84.7%)**	
**Patient characteristics**				
Age (years)^b^	59.0 (55.0–64.2)	60.00 (57.00–65.50)	59.00 (53.0–64.0)	0.403
Male	79.00%	85.20%	77.85%	0.546
Female	21.00%	14.80%	22.15	
Weight (kg)^a^	78.20 (15.0)	78.55 (12.89)	78.11 (15.44)	0.876
Height (cm)^a^	169.00 (8.92)	168.81 (8.37)	169.32 (9.05)	0.776
BMI (kg-m^2^)^a^	27.30 (4.68)	27.48 (3.83)	27.22 (4.83)	0.757
**Diagnoses and preoperative data**				
Indications for LT				
Alcoholic cirrhosis	58.00%	77.78%	54.36%	0.040
NASH	9.06%	7.41%	9.40%	1
Hepatocarcinome	9.66%	7.41%	10.70%	> 0.999
Biliary cirrhosis	7.39%	0%	8.72%	0.223
Other	15.89%	14.81%	17.45%	> 0.999
Prior abdominal surgery	32.40%	33.33%	32.21%	> 0.999
Diabetes	33.00%	22.22%	34.90%	0.286
Partial portal thrombosis	6.82%	18.52%	4.70%	0.022
Altered echocardiogram	16.50%	14.81%	16.78%	> 0.999
Pulmonary disease	17.6%	29.63%	15.44%	0.097
Ascites/pleural effusion	54.00%	66.67%	51.68%	0.219
Ascites volume (l)^b^	3400.00 [1500.00–6925.00]	3350.00 [1500.00–6550.00]	3400.00 [1450.00–6925.00]	
Preoperative kidney dysfunction	26.10%	40.74%	23.49%	0.107
Sodium (mmol.l^−1^)^b^	136.00 (131.00–139.00)	132.00 (129.00–136.00)	136.00 (131.00–139.00)	0.042
Creatinine (mg.kg^−1^)^b^	0.94 (0.76–1.22)	1.09 (0.83–1.40)	0.92 (0.75–1.20)	0.063
MELD score^b^	19.0 (13.0–23.0)	21.00 (17.50–26.50)	18.0 (13–22.0)	0.007
Child–Pugh score				0.216
A	15.50%	11.11%	16.33%	
B	33.90%	22.22%	36.05%	
C	50.60%	66.67%	47.62%	
UNOS classification				0.007
At home	56.25%	37.04%	59.73%	
On ward	34.09%	37.04%	33.56%	
ICU	9.66%	25.93%	6.71%	
Haemoglobin (g.l^−1^)^b^	93.00 (84.00–108.00)	92.00 (81.00–109.00)	103.00 (89.00–118.00)	0.019
Platelet count (× 10^–9^)^−1b^	74.00 (52.50–101.00)	68.00 (45.50–84.00)	103.00 (89.00–118.00)	0.123
PTT^b^	1.20 (1.06–1.36)	1.30 (1.16–1.53)	1.20 (1.04–1.35)	0.030
PT/INR^b^	1.55 (1.33–1.81)	1.77 (1.52–2.24)	1.52 (1.30–1.73)	0.004
Fibrinogen (g.l^−1^)^b^	2.00 (1.31–3.0)	1.54 (1.18–2.01)	2.15 (1.40–3.03)	0.006
ExTem				
Coagulation time (s)^b^	65.00 (59.00–75.00)	72.00 (62.00–86.50)	64.00 (58.75–73.25)	0.022
MCF (mm)^b^	51.00 (43.00–60.00)	72.00 (62.00–86.50)	72.00 (62.00–86.50)	0.020
Lysis^b^	0 (0–0)	0 (0–2)	0 (0–0)	0.008
A10 FibTem MCF (mm)^b^	11.00 (6.00–16.00)	7.00 (4.50–12.50)	11.00 (7.00–16.00)	0.016

*ExTem *Extrinsic thromboelastometry for fibrin tissue factor activation, *FibTem *Thromboelastometry for fibrin tissue, *LT* Liver transplantation, *MCF* Maxim clot firmness, *MELD* Model for End-Stage Liver Disease, *NASH* Nonalcoholic steatohepatitis, *PT* Prothrombin time, *PTT* Partial thromboplastin time, *PT/INR* International normalised ratio of PT, *RBC* Red blood cells, *UNOS* United network for organ sharing

Vena cava preservation was achieved in 96% of patients in both groups. Intraoperative venovenous bypass was used in four patients (three in the early extubation group and one in the prolonged mechanical ventilation group). A portocaval shunt was used in 65 (35.93%) vs. 12 patients (44.44%) (*p* = 0.508).

There were no differences in sociodemographic values or donor characteristics. Between-group differences were also detected in intraoperative data related to cold ischaemia time, postreperfusion syndrome, and use of tranexamic acid and blood products (Table 2). No differences in the use of intraoperative fluid therapy were found. However, more fluid therapy was used in the first 24 h after surgery in the group that required prolongation of mechanical ventilation.

**Table 2:** Patient surgical data. Data are number (%) or percentage of patients, unless otherwise indicated as mean (SD)^a^, median (IQR)^b^, or median (range)^c^

	**All** **(*n* = 176, 100%)**	**MV > 24 h** **(*n* = 27, 15.3%)**	**MV < 24 h** **(*n* = 149, 84.7%)**	***p* value**	
**Donor and intraoperative data**					
Donor type					
Brain death	68.20%	70.37%	67.90%	0.967	
Cardiac death	31.80%	29.63%	32.21%		
Donor age (years)^c^	59.00 (18.00–84.00)	58.00 (25.00–84.00)	60.00 (18.00–78.00)	0.89	
Length of surgery (min)^b^	390.00 (303.00–1436.00)	426.00 (325.00–1455.00)	380.00 (299.00–1430.00)	0.398	
CIT (min)^b^	373.00 (284.00–445.00)	400.00 (359.00–467.00)	356.00 (278.00–430.00)	0.014	
WIT (min)^b^	36.00 (26.00–50.00)	35.00 (28.50–45.00)	36.00 (26.00–50.00)	0.738	
Postreperfusion syndrome	46.60%	74.07%	41.61%	0.004	
**Transfusion During LT**					
RBC (units)^b^	2.00 (0.00–4.00)	3.00 (1.00–5.00)	1.00 (0.00–3.00)		0.001
RBC infusions					0.001
0 units	33.50%	7.41%	38.26%		
1–6 units	57.40%	70.37%	55.03%		
> 6 units	9.09%	22.22%	6.71%		
Fresh frozen plasma	12.50%	29.63%	9.40%		0.008
Apheresis platelets	13.64%	29.63%	10.74%		0.015
Tranexamic acid	39.20%	62.96%	33.56%		0.007
Crystalloids (ml)^b^	2280 (1228–3424)	2200 (1275–35,072)	2280 (1238–3200)		0.608
Albumin^b^	67.43%	88.89%	63.51%		0.018
Transfusion during and 24 h after LT					
RBC (units)	2.50 (0.00–5.00)	4.00 (2.00–7.00)	2.00 (0.00–4.00)	0.001	
RBC infusions				0.001	
0 units	26.10%	3.70%	30.20%		
1–6 units	55.70%	37.04%	95.06%		
> 6 units	18.20%	59.26%	10.74%		
Fluid therapy^a^ (ml)^b^	5234 (4153–7184)	6550 (4860–9055)	4962 (3951–6833)	0.004	

*CIT* Cold ischaemia time, *WIT* Warm ischaemia time

**Fig. 1:**
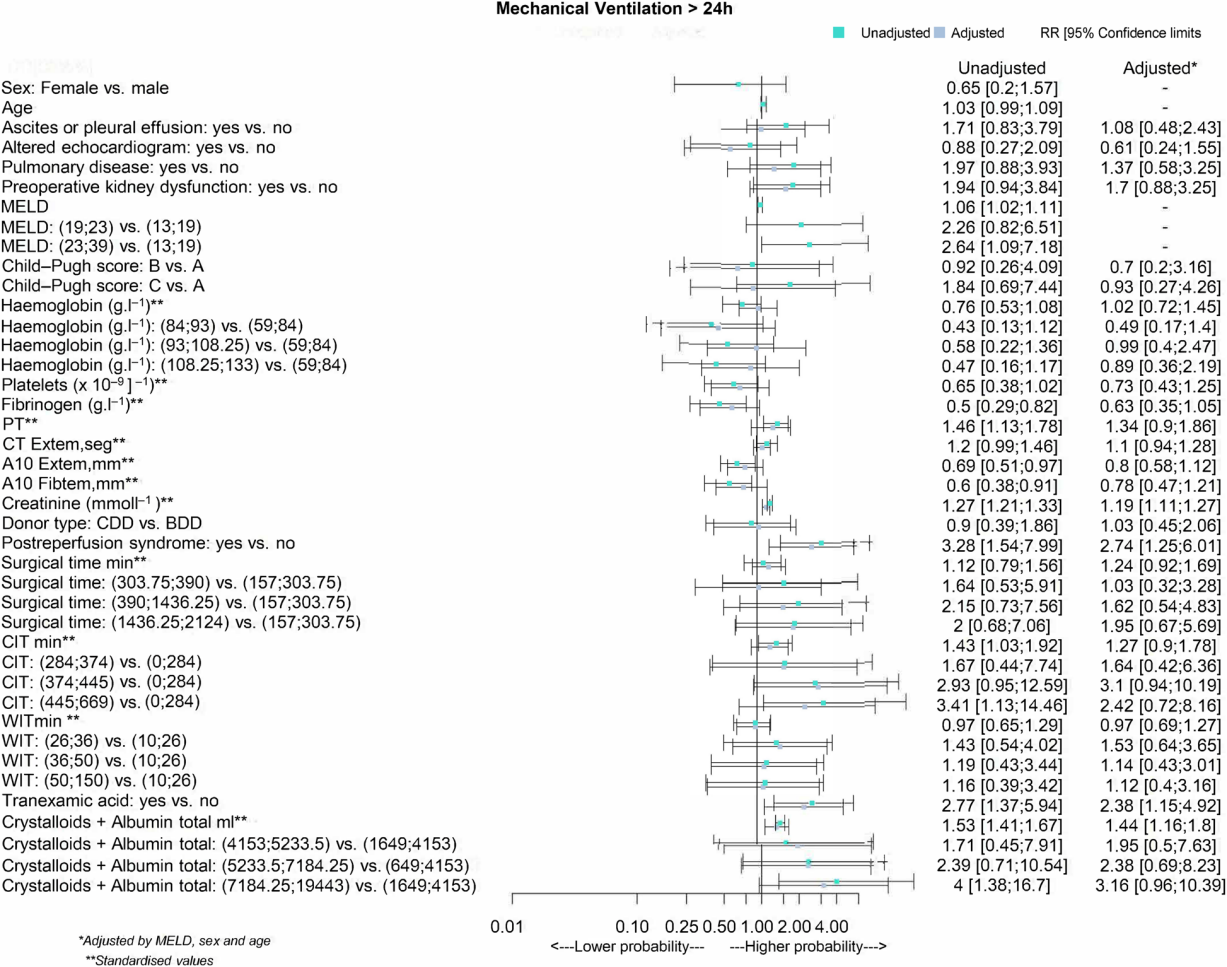
Relative risk of factors of all factors evaluated for association with prolonged mechanical ventilation (> 24 h) after liver transplantation. Upper and lower cut points for variable stratification are shown in parentheses in the first column. A grey square denotes unadjusted relative risk (RR); a blue square denotes RR adjusted by MELD score, sex and age. The whiskers indicate the 95% CI. BDD, brain-dead donors. CDD, cardiac-death donors. CIT, cold ischaemia time. CT, coagulation time. ExTem, extrinsic thromboelastometry for fibrin tissue factor activation. FibTem, thromboelastometry for fibrin tissue. MELD, Model for End-Stage Liver Disease. PT, prothrombin time. WIT, warm ischaemia time

In the regression model, age, sex, and donor type were not significantly related to prolongation of mechanical ventilation. However, MELD scores > 23 were significantly more common in patients who could not be extubated early. Once the model was adjusted for age, sex, MELD score, and donor type, the only variables that were significantly different in patients mechanical ventilated for > 24 h were the baseline creatinine value, the presence of postreperfusion syndrome, and the amounts of tranexamic acid and RBCs used (Fig. 1).

The median (IQR) stay in the postoperative ICU was 3 (2–4) days for patients in the extubation group < 24 h vs. 5 (3–10) days for those who required prolonged mechanical ventilation (*p* = 0.001). Eight patients in the extubation group (5.52%) were readmitted to the ICU. Nearly all of the ICU readmissions were related to reoperations. Only one patient (3.7%) in the prolonged-ventilation group was readmitted. The median length of hospital stay was 14 (10–24) days after early extubation and 25 (14–55) days after prolonged ventilation (*p* = 0.001).

## Discussion

Our study to identify modifiable preoperative and intraoperative risk factors associated with prolonged mechanical ventilation (> 24 h) found no relevant presurgical respiratory or cardiac risk factors. Associated intraoperative variables included blood product requirements, postreperfusion syndrome, and the use of tranexamic acid. In a recent series, high blood transfusion requirements during liver transplantation were significantly associated with the need for prolonged mechanical ventilation [11]. Even this association, we were unable to include the amount of blood products infused in the relative risk analysis because usage was very low in the early-extubated patients, nearly all of whom received < 2 units of RBCs. After graft reperfusion, the concurrent return of normal splanchnic circulation and the washout of preservation solution from the transplanted liver can lead to the severe haemodynamic disturbances of postreperfusion syndrome, and these events may in turn lead to major surgical bleeding and high blood product and tranexamic acid usage, which were risk factors for prolonged mechanical ventilation in our study. This observation is consistent with reports from retrospective series. [12, 13] These findings suggest to us the possibility that postreperfusion syndrome might be prevented by using vasopressor support strategies and infusing a bolus dose of 500 mg of tranexamic acid prior to graft reperfusion to cut potential fibrinolysis. A controlled trial, however, would be required to confirm this hypothesis.

Severity of liver disease (the preoperative MELD score) was the main, but non-modifiable, risk factor for late extubation in our series, supporting previously reports [4, 5]. All the patients requiring prolonged mechanical ventilation in our series, however, had much higher MELD scores (> 23). This is in concordance with a recent study, where a MELD score of > 22 was associated with longer mechanical ventilation [14]. In that study, however, surgical technique (venovenous bypass) was used in 20% of patients and was a risk factor for prolonged mechanical ventilation, whereas in our study, nearly all patients were managed with vena cava preservation, and only in four patients (2.27%) a venovenous bypass was required.

Donor type was not associated with prolonged ventilation, an unsurprising finding given that normothermic and hypothermic oxygenation perfusion machines currently improve graft viability after procurement. [15]

Once the model was adjusted for MELD score, only creatinine level was maintained as a preoperative risk factor for prolonged mechanical ventilation. No other baseline characteristics such as coagulation or thromboelastometry parameters remained relevant. Both, hepatopulmonary syndrome and pulmonary hypertension, were similar distributed in both groups, therefore did not influence early extubation.

One study assessed a large number of preoperative and intraoperative variables to select patients at risk for prolonged mechanical ventilation and developed a risk model that used a MELD cutoff of 12 or less in the equation, [16] indicating a much lower level of severity of liver disease than in our series. That model was applied in a clinical study to stratify patients for very early extubation. [5] However, besides the difference in disease severity between patients in our study and these previous ones, it is important that they were not designed to seek modifiable factors. In contrast, we generated separate relative risk assessments of all factors that could be modified during the surgery.

Early extubation was associated with a shorter ICU stay in our series overall, but it did not protect against ICU readmission. However, second readmissions were mostly related to reoperations, underlining the importance of careful vigilance of graft response to reperfusion and management of complications.

This was a post hoc analysis of a randomised controlled trial, where most of the data related to intraoperative homeostasis and haemostasis management. This is a possible limitation of our study. The main limitation of this study is related to the exclusion of 93 patients who were at low risk for transfusion (baseline haemoglobin levels > 130 g.l^−1^). These exclusions were necessary for the randomised controlled trial which provided the data [7]. However, the excluded patients also had also low MELD scores, whereas the median MELD score in patients included in our trial was 19, which is common in the majority of patients on waiting for liver grafts in European registries [17]. Strengths of the study are the participation of three hospitals with high volumes of liver transplantation, prospective data collection, on-time compliance with the short patient recruitment period in spite of the SARS-CoV-2 pandemic, high adherence to protocols, and the monitoring of data quality by an independent committee.

We conclude that a MELD score > 23, bleeding, and postreperfusion syndrome are the main factors associated with prolonged mechanical ventilation and longer ICU stays. Only intraoperative bleeding and postreperfusion syndrome allow for modifiable interventions. Our hypothesis would therefore be that to improve outcomes we should design trials to explore vasopressor support strategies and interventions to prevent potential fibrinolysis prior to reperfusion.

## Data Availability

The datasets used and/or analysed during the current study are available from the corresponding author on reasonable request.

## Abbreviations

CIT: Cold ischaemia time
ExTem: Extrinsic thromboelastometry for fibrin tissue factor activation
FibTem: Thromboelastometry for fibrin tissue
LT: Liver transplantation
MCF: Maxim clot firmness
MELD: Model for end-stage liver disease
NASH: Nonalcoholic steatohepatitis
PT: Prothrombin time
PTT: Partial thromboplastin time
PT/INR: International normalised ratio of PT
RBC: Red blood cells
UNOS: United network for organ sharing
WIT: Warm ischaemia time
